# Transurethral Electrovaporization of Bladder Diverticulum: An Alternative to Open or Laparoscopic Bladder Diverticulectomy

**DOI:** 10.1089/cren.2015.29002.cha

**Published:** 2015-10-01

**Authors:** Ryan A. Chandhoke, Gamal M. Ghoniem

**Affiliations:** Department of Urology, UC Irvine Health, Orange, California.

## Abstract

We used transurethral electrovaporization of the diverticular mucosa as the primary treatment for an acquired bladder diverticulum in a female patient. The bladder diverticulum was secondary to bladder outlet obstruction from a previous pubovaginal sling. In comparison to either open or laparoscopic bladder diverticulectomy, transurethral electrovaporization of the bladder diverticulum was effective in significantly reducing the diverticular size while being less invasive, requiring a short operative time, and a quick patient recovery.

## Clinical History

A 61-year-old woman presented to our institution in 2013 with complaints of incomplete bladder emptying, difficulty in urination with voiding only in a bent-over position, urinary frequency, and nocturia. She also had a history of recurrent urinary tract infections (UTI) and pelvic pain. Her urogynecologic surgical history included vaginal hysterectomy, rectocele repair with perineoplasty, placement of a pubovaginal sling with autologous fascia, and cystocele repair at an outside institution in 2001.

Other medical and surgical histories were unremarkable. Her social history included three glasses of wine at night and a former 10 pack year history of smoking.

## Physical Examination

The abdominal examination showed suprapubic tenderness with a palpable bladder and mild left lower quadrant tenderness. Pelvic examination showed stage one anterior prolapse, negative cough urinary stress test, and a zero degree Q-tip test without urethral hypermobility. The rectal examination was unremarkable.

## Diagnostic Studies

### Flexible cystoscopy

The urethral meatus and urethra were normal. There was a large left lateral wall diverticulum and moderate bladder trabeculation. The mouth of the diverticulum was wide and easily entered and showed a smooth endothelium without evidence of calculus, tumor, or foreign bodies.

### Fluorourodynamics

At a filling rate of 30 mL/second, normal compliance was noted. Her first desire and strong desire to void occurred at volumes of 187 and 249 mL, respectively. Urethral pressure profilometry showed a proximal and maximal urethral pressure of 20 and 103 cm of H_2_O, respectively. Functional urethral length measured 4.5 cm. The voiding phase showed a total voided volume of 327 mL with a maximum flow rate of 22 mL/second and a coinciding detrusor pressure of 43 cm of H_2_O.

**FIG. 1.**
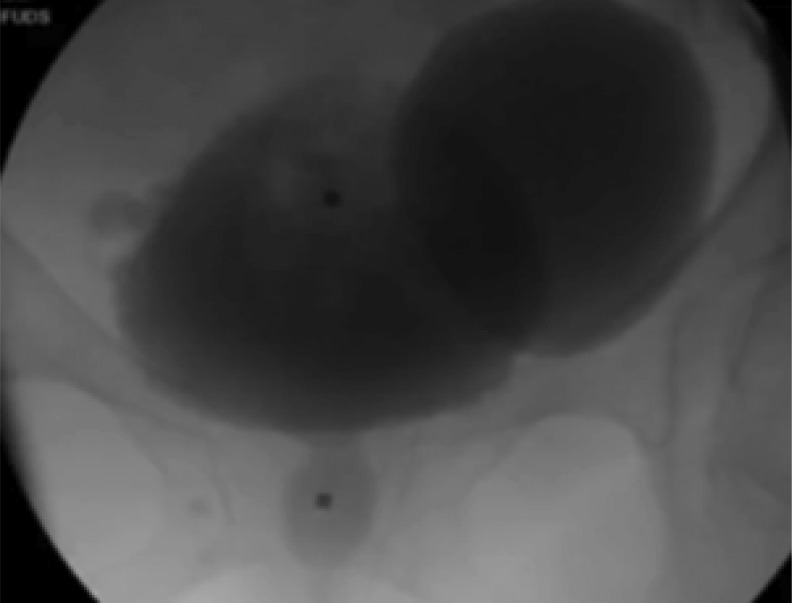
Preoperative voiding cystourethrogram (VCUG) showing ballooning of large left bladder diverticulum measuring 400 mL based on post void residual urine volume.

Fluoroscopic images taken during urodynamics showed the presence of a left large diverticulum without reflux and minimal urethral hypermobility. During voiding, there was ballooning of the diverticulum with retention of contrast (Fig. 1). The bladder emptied to completion while the diverticulum retained the contrast. After an additional few minutes, another image was taken which demonstrated that approximately half of the contrast from the bladder diverticulum had emptied back into the bladder.

The fluorourodynamic diagnosis was that of bladder outlet obstruction (BOO) with high-pressure high-flow voiding. The obstruction site was at the mid-urethral level. The bladder pressure may have been higher if not for the additional accommodation of the diverticulum. The diverticulum retained almost all the residual urine with a volume of ∼180 mL. Follow-up residual urine measurements showed higher volumes of 400 mL. Furthermore, diverticular ballooning during voiding showed that a significant amount of urine traveled into the diverticulum instead of being voiding out through the urethra. Subsequently, the bladder diverticular urine returned to the bladder causing her retention and incomplete emptying. The overall diverticular volume thus could be measured as the post void residual urine volume of 400 mL. BOO in the female population is rare and mostly iatrogenic; however, her diagnostic studies confirmed that the etiology was likely secondary to an obstructive pubovaginal sling.

## Intervention

In a patient with an acquired bladder diverticulum, the underlying BOO must be addressed first before any treatment of the diverticulum. Thus, the patient first underwent partial urethrolysis with excision of the autologous fascial sling. Her urination became easier and did not require changing position. Postoperatively, she continued to have incomplete bladder emptying and recurrent UTI. She then underwent an additional urethral dilation and subsequent urethral calibration showing no further evidence of BOO.

Treatment of the bladder diverticulum was now entertained. The patient was counseled on the treatment options of open and laparoscopic bladder diverticulectomy, as well as transurethral electrovaporization of the bladder diverticulum. The patient chose the latter because of its potential equal effectiveness to open and laparoscopic approaches while being less invasive with a quicker recovery time.

**FIG. 2.**
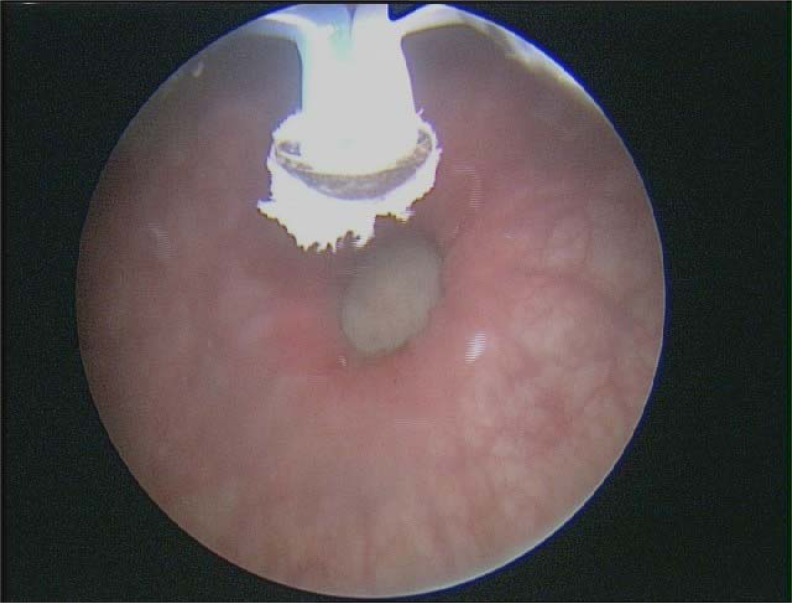
A button bipolar vaporization electrode outside the left bladder diverticulum opening.

The patient underwent rigid cystoscopy under general anesthesia that re-demonstrated the large left bladder diverticulum that could be accessed effectively with rigid instruments. Transurethral electrovaporization of the entire diverticular mucosa was then performed using a button vaporization electrode (Fig. 2). Bipolar settings for cutting and coagulation were 280 watts and 140 watts respectively. Only the coagulation setting was used. During electrovaporization, the intraluminal volume of the diverticulum visibly reduced in size. There was no evidence of bleeding, perforation, or ureteral orifice injury during the case. Total electrovaporization time lasted 30 minutes and the total operative time was 40 minutes. An indwelling urethral catheter was placed at the end of the case and left in for a total of 6 weeks. She was kept on a prophylactic daily dose of nitrofurantoin and discharged home on the same day of her surgery.

## Outcome

**FIG. 3.**
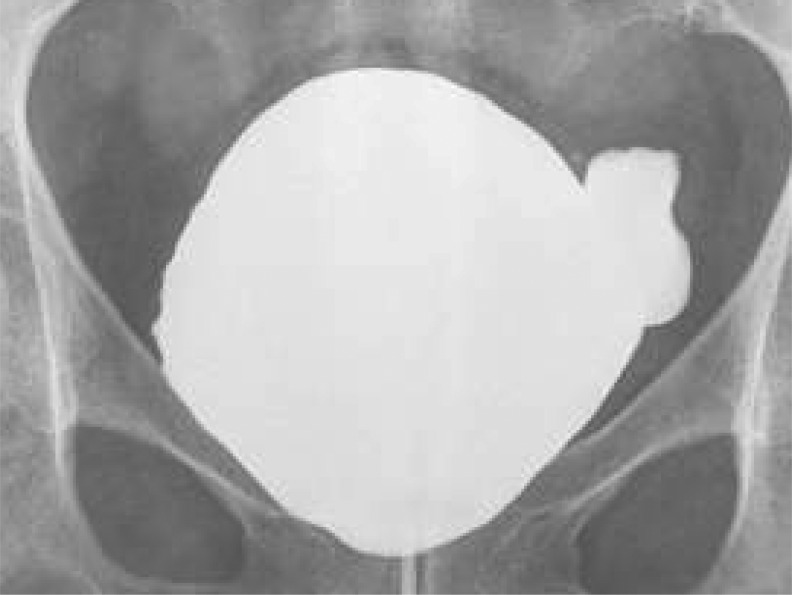
Postoperative VCUG showing significant reduction in diverticulum size at full bladder distension with minimal post void residual urine volume.

The patient did well postoperatively without any signs of UTI. A voiding cystourethrogram (VCUG) was performed first at 4 weeks and then at 6 weeks postoperatively. VCUG at 6 weeks showed a significant reduction in diverticulum size (Fig. 3). The Foley catheter was removed at 6 weeks, and the patient was able to void without difficulty with minimal residual urine and devoid of any recurrent UTIs.

Acquired bladder diverticulum is the result of BOO necessitating high-pressure voiding. After the correction of the BOO, the bladder diverticulum requires intervention in the presence of incomplete emptying, recurrent infection or pain; all of which this patient experienced. Diverticulectomy was first described in 1897.^1^ It has evolved from open surgery to laparoscopic and robotic techniques. Additionally, numerous reports demonstrate reproducibility with the transurethral approach.^2^

Our case shows a rare example of acquired large diverticulum in a female patient due to an obstructive pubovaginal sling. After sling removal and urethrolysis with subsequent urethral calibration, we were able to treat the large diverticulum with transurethral electrovaporization. Plasma vaporization is realized with near-close tissue contact, with minimal heat generation and excellent hemostasis. In either monopolar or bipolar technique for bladder diverticulum, perforation or injury is rare regardless of diverticular size. As opposed to monopolor technique, bipolar electricity does not have to travel through the body to the skin electrode to close the circuit. Bipolar tissue penetration is also much shorter, ranging from 50-100 μm, generating less collateral thermal damage and tissue charring.^3^ Additionally, bipolar technique avoids nervous stimulation and pacemaker dysfunction, making it a safer procedure. This minimally invasive technique along with the ease of surgery and faster operating time, with similar clinical outcome to open and laparoscopic diverticulectomy, exemplifies its usefulness in the clinical setting. All surgical options for the treatment of the bladder diverticulum, which require intervention, should be discussed with the patient during the preoperative counseling. This case report of transurethral electrovaporization of the bladder diverticulum is in accordance with previous case reports showing favorable outcomes of transurethral techniques for the surgical management of a bladder diverticulum.

## Abbreviations Used

BOO: bladder outlet obstruction
UTI: urinary-tract infections
VCUG: voiding cystourethrogram
